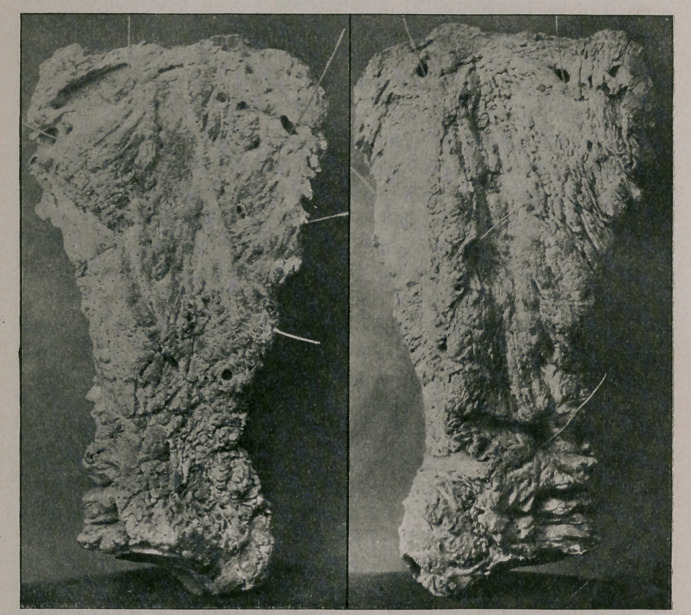# Necrosis Totalis of the Scapula

**Published:** 1897-02

**Authors:** J. D. Nighbert

**Affiliations:** Pittsfield, Ill.


### REPORTS OF CASES.

### NECROSIS TOTALIS OF THE SCAPULA.

By J. D. Nighbert, V.S.,
PITTSFIELD, ILL.

On August 1, 1889, I was called to see a valuable roadster stallion which, a few days previously, had been kicked on the point of the left shoulder. An examination disclosed a small wound of the integument and tissues at the lower point of the shoulder, near the termination of the spine; lameness excessive; temperature 103°; swelling of entire shoulder, but better marked around the point; a slight bloody discharge from the wound.

On exploration with a probe found the lower point of process fractured as well as the outer plate of the neck of scapula. Removed several thin pieces of bone, ordered hot applications to the entire shoulder, and thorough cleansing of wound three times a day with bichloride solution.

Internal treatment consisted of two-drachm doses of potassium nitrate twice daily in drinking-water and rectal injections of warm water; this treatment was continued for six days. Swelling and temperature kept increasing; discharge from wound fetid.

On seventh day fluctuating swelling just back of wound, which, being freely opened, several ounces of very fetid pus escaped. From the general swelling of the entire shoulder and the character of discharge a diagnosis of total necrosis of scapula was made and an unfavorable prognosis was given; but as the horse was a valuable one owner thought best to continue treatment, as the horse might sufficiently recover to be of some use for breeding purposes. After thoroughly cleansing cavity I inserted drainage-tube and continued washing three times a day with the bichloride solution. This was followed by a subsidence of swelling where abscess was opened; but near the cervical angle a like abscess formed and was opened and treated as the previous one, followed by a slight decrease of swelling, but pus continued to flow freely from all the openings. In the course of four or five days a like swelling appeared in the region of the dorsal angle. Opened and treated as previous one. This was followed by another abscess in the region of the tuberosity of the spine. The cause of these pus-centres will be readily seen by referring to the illustration of the external and internal surfaces of scapula, where are large cloacæ, exposing the sequestrum, which is almost covered by the sequestral capsule.

The horse now began to show some improvement, but great quantities of pus continued to flow from all the openings. Being now able to walk some, owner thought he would take him to the country, two or three miles out, and care for him himself. In the course of ten or twelve days the wounds almost healed, leaving small, fistulous openings which did not permit the pus to discharge freely enough, and there appeared a swelling in the region of the lower third of the ribs just back of the elbow. This swelling gradually extended to sheath, and owner thought that it needed opening, so he plunged a knife in the softest place just back of the elbow, getting a copious flow of pus, but unluckily severing a large branch of the external thoracic artery. I was hurriedly sent for, and seeing that further treatment was useless I advised his destruction, and, securing the scapula, had it cleansed, and herewith present photograph of the two surfaces, showing the remarkable deposit of lime-salts accumulating in the space of twelve weeks.

## Figures and Tables

**Figure f1:**